# Comparisons of Corn Stover Silages after Fresh- or Ripe-Corn Harvested: Effects on Digestibility and Rumen Fermentation in Growing Beef Cattle

**DOI:** 10.3390/ani12101248

**Published:** 2022-05-13

**Authors:** Min Zhang, Rong Wang, Tingting Wu, Yingbai Yang, Zhixiong He, Zhiyuan Ma, Zhiliang Tan, Bo Lin, Min Wang

**Affiliations:** 1 College of Animal Science and Technology, Guangxi University, Nanning 530004, China; 2 Key Laboratory for Agro-Ecological Processes in Subtropical Region/National Engineering Laboratory for Pollution Control and Waste Utilization in Livestock and Poultry Production, Institute of Subtropical Agriculture, Chinese Academy of Sciences, Changsha 410125, China

**Keywords:** harvest time, fiber digestibility, microbiota, rumen fermentation, utilization of agricultural byproducts

## Abstract

**Simple Summary:**

Corn stover is an important agricultural byproduct, and represents an animal feedstuff. Waxy corn is harvested at milk stage or dough stage (20 d difference in harvest time) to meet the market demand for fresh corn and ripe corn in China, resulting in plenty of byproducts of corn stover after fresh- (CF) or ripe-corn (CR) harvested. This study was conducted to investigate the digestibility and rumen fermentation of these two corn stover silages in growing beef cattle. We observed that CR silage had greater dry matter and fiber contents, but with similar substrate degradation through in vitro ruminal fermentation in comparison with the CF silage. Further feeding trials indicated that feeding a CR diet (i.e., replaced 50% forage of CF silage with CR silage on a dry matter basis) increased feed intake and decreased fiber digestibility with reduction in the abundance of ruminal fiber degraders, together with similar growth performance in beef cattle, when comparing with CF diet.

**Abstract:**

Both waxy corn stover after fresh- (CF) and ripe-corn (CR) harvested are important byproducts of corn cropping system and have 20 d difference in harvest time. The study aimed to investigate the effects of prolonging harvest time on the nutritive value of corn stover silage by comparing CF with CR silages. In vitro ruminal experiment was firstly performed to investigate substrate degradation and fermentation of CF and CR silages. The CR diet was formulated by replacing 50% forage of CF silage with CR silage on a dry matter (DM) basis. Fourteen crossbred steers (Simmental × Limousin × local Chinese) aged 13 months with an average weight of 318.1 ± 37.1 kg were selected and randomly allocated into two dietary treatment groups. Although the CR silage had greater DM and fiber contents than CF silage, it did not alter in vitro degradation (*p* > 0.05), but with lower molar percentage of propionate and acetate to propionate ratio (*p* < 0.05). The cattle fed CR diet had a higher DM intake and lower fiber digestibility with reduction in 18S rRNA gene copies of protozoa and fungi and 16S rRNA gene copies of *Fibrobacter succinogenes* (*p* < 0.05). Further 16S rRNA gene amplicon analysis indicated a similar diversity of bacteria community between CR and CF treatments (*p* > 0.05). Few differences were observed in the abundance of genera larger than 1% (*p* > 0.05), except for the reduction in abundance of genera *Ruminococcaceae*_NK4A214_group in CR treatment (*p* < 0.05). In summary, prolonging 20 d harvest time of corn stover silage increases the forage fiber and DM content, which promotes feed intake with decreased fiber degradation, although rumen fermentation and growth performance are not changed in growing beef cattle.

## 1. Introduction

Corn (*Zea mays*) is an important cash crop, and China is the second largest corn producer in the world [1]. Waxy corn (*Zea mays* L. *sinensis Kulesh*), widely grown in East and Southeast Asia, can be harvested as fresh vegetable at milk stage (fresh-corn) or as starch extraction at dough stage (ripe-corn) [2,3,4]. These different cropping systems can result in two types of corn byproducts, i.e., corn stover after fresh- or ripe-corn harvested, which can be utilized to produce silage for ruminant animals. Choosing the suitable cropping system according to the price of corn in the market can effectively improve the economic interests of cropping farmers, and efficient utilization of crop byproducts is beneficial to the development of the circular economy balanced with environment and resource [1].

Harvest time is an important factor to influence the nutrient composition of herbage [5,6]. Prolonging harvest can increase fiber content and lignification in its stems and leaves of corn as it grows like many grasses [7,8,9]. It is hard for ruminants to digest lignified fiber [10], so the greater lignified fiber in forage is detrimental to its application in ruminants. Meanwhile, prolonging harvest can also increase the dry matter (DM) content of corn stover [8], which is important to maintain quality of silage [11]. The difference of harvest time is about 20 d between corn stover after fresh-corn harvested (CF) and corn stover after ripe-corn harvested (CR). There is still a lack of study to investigate effects of these two types of silage on digestibility and rumen fermentation in ruminant production.

This study was then designed to investigate if prolonging the harvest time of 20 d could alter the nutritive value of corn stover silage and its consequence on digestibility, rumen fermentation, and growth performance in growing beef cattle. In vitro batch cultures were firstly performed to compare the ruminal fiber degradation and fermentation of CF and CR silage. Once it was established which fiber source was more digestible, an in vivo study was then performed to evaluate the effect of altering dietary forages by substituting CF with CR silage on fiber digestibility, rumen fermentation, and microbiota in growing beef cattle.

## 2. Materials and Methods

### 2.1. Preparation of Corn Stover Silages

We first obtained silages of corn stover after fresh-corn harvested (CF) and corn stover after ripe-corn harvested (CR). The waxy corn (*Zea mays* L. *sinensis Kulesh*) was planted in an area of about 2 hectares by local farmers in Nanning City, Guangxi, China (22°70′ N, 108°03′ E). Each plot of this area was divided into two halves in the middle and one half of corn was harvested at random at a time. Fresh corn was harvested on 14 September 2018 when 50% to 60% of the grain has the characteristics of milk stage, while ripe corn was harvested on 4 October 2018 when 80% of grain has the characteristics of dough stage. The CF and CR were collected with the stem cut about 15 cm above the ground level. These two types of corn stover were ensiled for 2 months in plastic bags with 45 kg per bag by corn stover baler (Baler 230, Jining Deruisi Mechanical Technology Co. Ltd., Jining, China). The samples were collected from the middle and sides of the bags. The subsample was prepared by balanced mixing of all samples (4 sampling weeks × 3 bags) and dried at 65 °C for subsequent in vitro incubation. Dry matter (DM) content and chemical composition, including organic matter (OM), crude protein (CP), neutral detergent fiber (NDF), acid detergent fiber (ADF), starch, and gross energy (GE), were then measured and showed in Table 1.

### 2.2. In Vitro Incubation

The subsample was shattered by a pulverizer and then screened through a 1-mm stainless steel flour sieve. About 600 mg of substrate was weighed and put into the serum bottles, followed by 60 mL of artificial rumen fluid mixed by rumen fluid and artificial saliva at the ratio of 1:4 [12] under a continuous flow of carbon dioxide at 39.5 °C. A fully automatic in vitro batch system was employed to investigate the rumen fermentation characteristics of these two silages [12], and the artificial saliva added was formulated according to Menke et al. [13]. The fermentation was stopped after 48 h of incubation to measuring substrate degradation and fermentation end-products. Undegraded residues after incubation were collected to measure chemical composition, including DM, NDF, and ADF. The fermented liquid was immediately collected to measure the pH value with a portable pH meter (Starter 300; Ohaus Instruments Co., Ltd., Shanghai, China). Each bottle was shaken by hand to collect 2 mL of sample, which was then centrifuged at 12,000× *g* for 10 min, and the supernatant was stored at −20 °C for the measurement of volatile fatty acid (VFA) concentration. Three runs of incubation were conducted. Two replicates per substrate were included in each run of incubation.

### 2.3. Animal Trial

#### 2.3.1. Cattle and Diets

The animal trial was conducted at the beef cattle farm of Guangxi Huisheng animal husbandry development Co., Ltd., Long’an county, Nanning city, China. Fourteen crossbred cattle steers (Simmental × Limousin × local Chinese) aged 13 months with an average weight of 318.1 ± 37.1 kg were selected and randomly allocated into two dietary treatment groups (7 cattle per group). In the preliminary experiment, body weight (BW) and dry matter intake (DMI) were recorded for subsequent allocation of animals to 7 blocks. Every two animals were assigned to 1 block, and one animal within each block was fed with 1 of 2 diets randomly. The CR diet was formulated by replacing 50% forage of CF with CR silage (DM basis). The diets were formulated to meet appropriately 1.2 to 1.3 times energy and protein requirements of growing beef bulls by referring Zhang and Zhang [14] (Table 2).

All the cattle were kept in a tie-stall barn and were fed twice a day at 7:00 am and 4:00 pm with free access to total mixed ration (TMR) and water. The experimental period included 10 d as a pretrial period to obtain a suitable amount to be fed after that and 60 d as a formal experimental period. The last 10 d was employed for sampling and data collection, including 5 d for total feces collection, 2 d for collecting rumen samples, and 3 d for measuring BW. Feed intake was estimated during the initial 10 d of the adaption. To minimize diet selection, the offered and refused feed were weighed and recorded on a daily basis, and the feed allowance was adjusted biweekly according to previous DMI after allowing an extra refusal amount of approximately 5% of the daily DMI. The average daily gain (ADG) was calculated as BW gain divided by the number of experimental days.

#### 2.3.2. Nutrient Digestibility

Feeds, refusals, and fecal samples were collected daily from d 61 to 65. Total feces were collected every 4 h per day, weighed and mixed daily. Approximately 5% of the total fresh weight of the fecal samples were sampled in duplicate after mixing with a stick. Fresh fecal subsamples (1%, *w*/*w*) were acidified with 10% (*v*/*v*) sulfuric acid for protein content analysis [16], while non-acidified fecal subsamples (1%, *w*/*w*) were collected for the rest of the chemical analyses. Fecal subsamples were placed in a refrigerator at −20 °C immediately to limit the multiply of microbes. All the samples were placed in a forced-air oven at 65 °C for 24 h for primary drying, and then shattered by a 1 mm screen for subsequent analysis of chemical fractions, including DM content, NDF, ADF, CP, starch, and GE.

#### 2.3.3. Rumen Sampling

Rumen contents were collected at 0 and 2.5 h relative to the morning feeding on d 66 and 67. Approximately 500 mL of rumen content was collected from the middle part of the rumen through a stomach tube using the method described by Wang et al. [17]. The pH of the rumen contents was detected with a portable pH meter immediately by separating an about 20 mL of sample. Two 35 mL samples were used to measure dissolved hydrogen and methane as described by Wang et al. [17]. Three 5 mL samples were centrifuged at 12,000× *g* for 10 min, and the supernatant was stored at −20 °C for measuring ammonia-N and individual VFA concentration. About 50 mL of rumen content collected at 0 h relative to morning feeding was immediately frozen in liquid N_2_ and stored at −80 °C for DNA extraction and microbial analyses.

### 2.4. Sample Analysis

#### 2.4.1. Chemical Composition

The DM content of samples was determined by placing it in an oven at 105 °C for 24 h. The OM (method 942.05) and CP (method 970.22) were determined according to methods of AOAC. The NDF and ADF were measured by a Fibretherm analyzer (C. Gerhardt Inc., Königswinter, Germany) following the procedures of Van Soest et al. [18] and expressed as inclusive of residual ash with inclusion of α-amylase (Sigma, Shanghai, China) for NDF. GE was determined using an isothermal automatic calorimeter (5E-AC8018; Changsha Kaiyuan Instruments Co., Changsha, Hunan, China). Starch content was determined after pre-extraction with ethanol (80%), and glucose released from starch by enzyme hydrolysis was measured using amyloglucosidase [19].

#### 2.4.2. Fermentation Parameters

The acidified rumen samples were re-centrifuged at 15,000× *g*, and the VFA in the supernatants were analyzed with gas-liquid chromatography (Agilent 7890, Palo Alto, CA, USA) [19]. Estimated net hydrogen production relative to the amount of total VFA produced was calculated based on the VFA profile [20]. Ruminal ammonia-N concentration was measured using phenol-hypochlorite reaction [21].

Dissolved gas concentration was measured after building a new equilibration between gas and liquid phase for the samples collected in 50 mL plastic syringes using pure N_2_ gas (>99.99%) [22]. The extract hydrogen and methane concentrations in the gas phase were measured by gas chromatography (Agilent 7890A, Agilent Inc., Palo Alto, CA, USA). Concentrations of dissolved hydrogen and methane in the original liquid fraction were calculated using equations described in Wang et al. [22].

#### 2.4.3. DNA Extraction and Microbial Analysis

Total genomic DNA from ruminal content samples (2 mL) was extracted using repeated bead beating plus column purification [23] and eluted by 300 μL TE buffer (Tris 10 mmol/L, EDTA 1 mmol/L, pH = 8.0). The quality and quantity of DNA were measured based on absorbance at 260 and 280 nm using a NanoDrop ND-2000 (NanoDrop Technologies Inc., Wilmington, NC, USA).

Quantitative real-time PCR (qPCR) was performed according to the procedures described by Jiao et al. [24]. Briefly, standard curves were made for selected microbial groups using plasmid DNA containing exact 16S or18S rRNA gene inserts and met the requirements as R^2^ > 0.99 and 90% < E < 120%. The microorganisms, including protozoa, fungi, bacteria, methanogens, *Fibrobacter succinogenes*, *Ruminococcus albus*, and *Ruminococcus flavefaciens*, were selected for their contribution to fiber digestion [10]. Forward and reverse primers of the selected microbial groups are shown in Table 3. The abundance of these microorganisms was calculated as copy numbers per mL of rumen content [25], and were further converted to log_10_ for further statistical analysis.

The 16S rRNA gene amplicons used for sequencing were described in previous teamwork [31]. Sequencing was done on Illumina MiSeq platform (Majorbio BioPham Technology, Shanghai, China). The sequencing readers were quality-controlled described by Kozich et al. [32] and classified into operational taxonomic units (OTUs) based on 97% similarity principle. The output data were normalized according to the smallest number of reads of the samples. Taxonomic analysis was carried out using the RDP classifier v.11.1 [33]. A principal coordinate analysis (PCoA) at the genus level was performed based on Bray–Curtis similarity distances [34]. Alpha diversity analyses were generated by MOTHUR v.1.39.5 [35] based on OTU, including Chao1, Species, Shannon, and Coverage indices.

### 2.5. Statistical Analysis

Data were subjected to analysis using the SPSS 21.0. The in vitro data were analyzed using the procedure of generalized linear model (GLM) that included dietary treatment (n = 2) as fixed effect, and incubation run (j = 3) as random effects. The in vivo data were analyzed using the procedure of GLM that included dietary treatment (n = 2) as fixed effect, and block (j = 7) as random effects. When sampling time was included, data were analyzed using a linear mixed model with treatment as fixed effect, block as random effect, and sampling time as a repeated measurement. Relative abundances of bacteria were commonly deemed non-normal, Wilcoxon rank sum tests were used to determine differences. A non-parametric permutational multivariate ANOVA (PMANOVA) test on the Bray–Curtis dissimilarity matrix, implemented in the vegan v2.5 (R package) was used to test the dietary effects on overall community composition. Significance was considered when *p* ≤ 0.05.

## 3. Results

The DM content, NDF, and ADF in CR silage were higher than those in CF silage, and CP and starch were similar (Table 1). Both CF and CR silages had similar DM, NDF, and ADF degradation (*p* > 0.05) with pH larger than 6.30 (Table 4). The CR silage had greater total VFA concentration with lower molar percentage of propionate and acetate to propionate ratio than that of CF silage (*p* < 0.05).

Cattle fed with CR diet had greater DM, OM, NDF, ADF, and starch intake and lower total-tract apparent DM, OM, and NDF digestibility, when compared with these fed CF diet (*p* < 0.05) (Table 5). The ADG was similar for two treatments (*p* > 0.05).

Cattle fed CR diet had similar (*p* > 0.05) ruminal dissolved hydrogen and methane concentration, ammonia-N, total VFA concentration, estimated net hydrogen production relative to the amount of total VFA produced and molar percentage of acetate, propionate, butyrate, valerate and isovalerate, and acetate to propionate ratio as these fed CF diet (Table 6). The time of sampling relative to feeding had a significant (*p* < 0.001) influence on the concentrations of dissolved hydrogen and methane, VFA and estimated net hydrogen production relative to the amount of total VFA. Rumen fluid collected at 2.5 h after morning feeding had greater ammonia-N, VFA concentration, molar percentage of propionate and valerate, and lower dissolved methane, pH, molar percentage of acetate and isobutyrate, and acetate to propionate ratio than these collected at 0 h (*p* < 0.05). Diet × time interaction had insignificant effect (*p* > 0.05) on most parameters, except pH and ammonia-N (*p* < 0.05).

**Figure 1:**
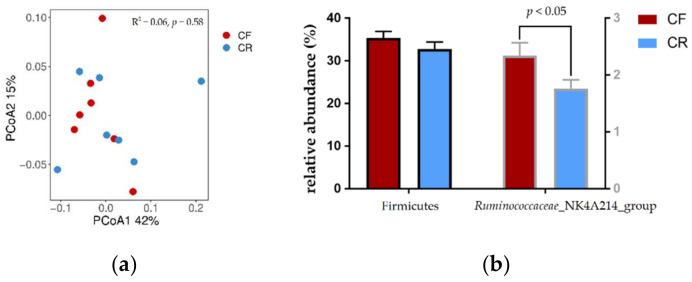
Bacterial community analysis based on the 16S rRNA gene sequencing. (**a**) The principal coordinate analysis (PCoA) of bacterial community based on Bray–Curtis dissimilarity matrix at the genus level. *R*^2^ = 0.06, *p* = 0.58. (**b**) The selected bacterial phyla and genera with significant differed between treatments, while other were present in Table S1. CF, silage diet with corn stover after fresh-corn harvested; CR, silage diet with corn stover after ripe-corn harvested.

Cattle fed CR diet had lower log 18S rRNA gene copies of protozoa and fungi, and log 16S rRNA gene copies of *F. succinogenes* by quantitative PCR analysis (*p* < 0.05) (Table 7). However, dietary treatment did not greatly alter bacteria community based on 16S rRNA gene amplicons sequencing with similar alpha diversity indexes (Chao1, Species, Shannon and Coverage) ((Table 8; *p* > 0.05). The principal coordinate 1 and principal coordinate 2 explained 42% and 15% of variation in bacterial genus, respectively, and the score plot did not show a distinct clustering of cattle fed CR or CF diet (Figure 1; *p* > 0.05). Few differences were observed in the abundance of genera with >1% abundance (Table S1; Figure 1). Dietary CR treatment increase abundance of genera *Ruminococcaceae*_NK4A214_group (*p* < 0.05), although no difference was observed in phylum Firmicutes (*p* > 0.05).

## 4. Discussion

The nutrients in a forage vary as it grows both in part and in whole, so there is an optimal harvest stage for forage in livestock feeding [7,36]. Khalilian et al. [37] have investigated the feeding value of seven sorghum varieties at different harvest stages, and the results showed that prolonging harvest increases DM yield and fiber content, which was consistent with our results. However, both CF and CR silage had similar in vitro substrate degradation, indicating that 20 d of prolonging harvest did not affect feed degradation. Nazli et al. [8] compared the nutritional value of four varieties corn silages in Malaysia, and there was no difference in digestible DM, digestible energy and metabolizable energy of corn silages reaped at milk and dough stage.

Beef cattle fed with the CR diet had greater DMI than those fed the CF diet. Cabezas-Garcia et al. [38] also reports that late-harvested grass silage had DM content and greater DMI than early-harvested grass silage in dairy cows. Mc Geough et al. [39] reported that corn silage harvested on 28 September had greater DMI than that harvested on 12 September. Such increased DMI could be caused by higher DM content in the silages of prolonging harvested forage, as two treatments had similar fresh weight intake. It has been widely reported that increasing feed intake will cause a reduction in apparent digestibility, especially in cases of when intake is higher than maintenance [40,41]. In our study, beef cattle fed CR diet had lower nutrient digestibility than these fed CF diet, indicating lower fiber utilization for the late-harvested silage. Such results were in agreement with Cabezas-Garcia et al. [38], which reported that part of the reduction in diet digestibility with increasing proportion of late-harvested grass silage can be related to increased passage rate with higher DMI. Depression in digestibility has been also reported for the diets containing alfalfa hay with increased feeding level [42]. Although two silages exhibited similar in vitro ruminal degradation, the decreased nutrient digestibility can be attributed to the greater feed intake in cattle fed CR diet, resulting in similar growth performance for two treatments.

Prolonging harvest increases the fiber content of forage, which can alter rumen fermentation profile by facilitating acetate production [43]. Our in vitro fermentation also indicated that the CR silage had greater molar percentage of acetate and acetate to propionate ratio than CF silage. However, this was not the case in vivo, and we found a similar molar proportion of major individual VFAs in cattle fed with CR or CF diet, which could be caused by the difference in feed intake. Lage et al. [44] reports that increasing feed intake decreases the molar percentage of acetate in the rumen of dairy heifers. Another study reported that elevated feed intake increases the molar percentage of propionate and decreases the molar ratio of acetate to propionate in bulls [45]. It seemed that increasing DMI facilitates propionate production by increasing passage rate [46], which can offset the promotion of acetate production caused by increased fiber content in CR silage. It has been reported that ruminal dissolved hydrogen and methane concentration always exhibits a strong association with rumen fermentation and microorganisms [47]. Such similar fermentation pathway was further upheld by the insignificant differences in ruminal dissolved hydrogen and methane with similar estimated net hydrogen production relative to the amount of total VFA produced [48,49].

The ability of ruminants to digest and ferment cell-wall biomass polymers strongly depends on the multitudinous microorganisms that reside in the rumen ecosystem [50]. Rumen fungi and protozoa are thought to play a role in the initial degradation of large fragments, which help the subsequent bacterial exposure to plant cell-wall polysaccharides [10,51]. In this study, cattle fed CR diet had fewer 18S rRNA gene copies of protozoa and fungi, and 16S rRNA gene copies of *F. succinogenes* in rumen, which was consistent with decreased NDF degradation. A study of fungi and protozoa in muskoxen rumen by a metatranscriptomic approach showed that these eukaryotes express large amounts of exo-glucanases to digest crystalline cellulose of forage [52]. Raut et al. [53] deciphered the cellulose degradation mechanism of the *F. succinogenes* S85, an efficient lignocellulose degrader isolated from the rumen of herbivores. Methanogens are major hydrogen-users in the rumen and its abundance was similar between two treatments, which were in agreement with unchanged ruminal dissolved hydrogen methane concentrations.

Change in bacterial community composition was further analyzed using high-throughput 16S rRNA gene sequencing. Cattle fed CR diet had similar species richness and diversity in terms of Chao 1 and Shannon with these fed CF diet, together with indistinct ruminal bacterial community between two treatments. These results illustrated that corn stover silage with prolonging harvest did not cause changes in ruminal bacterial community composition in beef cattle. An interesting result was that genus *Ruminococcaceae*_NK4A214_group, which could participate in fiber degradation in rumen [54], was less abundant with CR treatment, which was also consistent with the decreased fiber digestibility in these cattle.

## 5. Conclusions

Prolonging harvest of 20 d increased DM and fiber content of corn stover silage with decreased ruminal acetate to propionate ratio, but did not alter in vitro substrate degradation. Feeding CR diet increased feed intake and decreased nutrient digestibility than feeding CF diet, but did not change fermentation pattern in the rumen. The decreased fiber degradation in cattle fed CR diet can be attributed be decreased fibrolytic microorganisms, such as protozoa, fungi, and *F. succinogenes*, although rumen bacterial community composition was not greatly changed. Although 20 d of prolonging harvest altered the chemical composition and ruminal carbohydrate degradation, replacing up to 50% forage of CF silage with CR silage did not alter growth performance of growing beef cattle, which could be valuable for efficient utilization of these two types of corn-cropping byproducts in ruminant production.

## Figures and Tables

**Table 1:** Chemical composition of corn stover after fresh-corn harvested (CF) or corn stover after ripe-corn harvested (CR) silage.

Items	Silages	
	CF	CR
DM ^1^ (g/kg)	232	249
Substrate on a DM basis (g/kg DM)		
OM ^1^	928	926
CP ^1^	60	59
NDF ^1^	539	615
ADF ^1^	287	366
Starch	78	78
GE ^1^ (MJ/kg DM)	17.5	16.7

^1^ DM, dry matter; OM, organic matter; CP, crude protein; NDF, neutral detergent fiber; ADF, acid neutral detergent fiber; GE, gross energy.

**Table 2:** Ingredients and chemical composition of corn stover after fresh-corn harvested (CF) and corn stover after ripe-corn harvested (CR) silage diets.

Items	Diets	
	CF	CR
Dietary ingredient (g/kg DM ^1^)		
CF silage	530	265
CR silage	0	265
Maize bran	304	304
Wheat bran	56	56
Soybean meal	85	85
Limestone powder	5	5
* Saccharomyces cerevisiae*	10	10
NaCl	5	5
Premix ^2^	5	5
Chemical composition (g/kg DM)		
OM ^1^	922	921
CP ^1^	125	118
NDF ^1^	375	400
ADF ^1^	161	183
Starch	210	210
NEm ^3^ (MJ/kg DM)	6.78	6.53
NEf ^3^ (MJ/kg DM)	4.23	3.98

^1^ DM, dry matter; OM, organic matter; CP, crude protein; NDF, neutral detergent fiber; ADF, acid neutral detergent fiber; the DM content of CF diet was 35.7%, the DM content of CR diet was 36.7%. ^2^ The per kg DM of premix contained vitamin A (1,000,000 IU), vitamin D (200,000 IU), vitamin E (1250 IU), Zn (8000 mg), Co (40 mg), Se (80 mg), I (120 mg), Fe (2000 mg), Cu (2000 mg), and Mn (2500 mg). ^3^ Net energy for maintenance (NE_m_) and fattening (NE_f_) were calculated from gross energy as described by Feng [15].

**Table 3:** The primers (forward and reverse) of the selected microbial groups for qPCR.

Microbial Groups	Sequence ^1^ (5′→3′)	Reference
Bacteria	F: ACTCCTACGGGAGGCAGCA R: GGACTACHVGGGTWTCTAAT	[26]
Protozoa	F: GCTTTCGWTGGTAGTGTATT R: CTTGCCCTCYAATCGTWCT	[27]
Methanogens	F: GGATTAGATACCCSGGTAGT R: GTTGARTCCAATTAAACCGCA	[28]
Fungi	F: GAGGAAGTAAAAGTCGTAACAAGGTTTC R: CAAATTCACAAAGGGTAGGATGATT	[29]
Selected groups of bacteria		
* Ruminococcus albus*	F: CCCTAAAAGCAGTCTTAGTTCG R: CCTCCTTGCGGTTAGAACA	[30]
* Ruminococcus flavefaciens*	F: GAACGGAGATAATTTGAGTTTACTTAGG R: CGGTCTCTGTATGTTATGAGGTATTACC	[29]
* Fibrobacter succinogenes*	F: GTTCGGAATTACTGGGCGTAAA R: CGCCTGCCCCTGAACTATC	[29]

^1^ F = forward; R = reverse.

**Table 4:** The fermentation end-products for silage of corn stover after fresh-corn harvested (CF) and corn stover after ripe-corn harvested (CR) after 48 h in vitro ruminal incubation.

Items	Silages		SEM	*p*-Value
	CF	CR		
Substrate degradation (g/kg)				
DM ^1^	575	585	10.7	0.64
NDF ^1^	535	558	10.0	0.12
ADF ^1^	503	511	5.3	0.39
pH	6.49	6.61	0.016	0.003
Total VFAs ^1^ (mM)	82.3	87.7	3.80	0.02
Molar percentage of individual VFA (mol/100 mol)				
Acetate	63.4	64.1	1.38	0.60
Propionate	22.4	21.1	0.29	0.046
Butyrate	9.0	10.5	0.88	0.21
Valerate	1.2	1.2	0.18	0.84
Isobutyrate	1.2	1.2	0.12	0.91
Isovalerate	2.0	1.9	0.27	0.75
Acetate to propionate ratio	2.83	3.04	0.03	0.003

^1^ DM, dry matter; NDF, neutral detergent fiber; ADF, acid detergent fiber; VFA, volatile fatty acids.

**Table 5:** Feed intake and digestibility and growth performance in beef cattle fed corn stover after fresh-corn harvested (CF) or corn stover after ripe-corn harvested (CR) silage diet (n = 7).

Items	Diets		SEM	*p*-Value
	CF	CR		
Intake (kg/d)				
DM ^1^	7.47	7.97	0.210	0.04
OM ^1^	6.90	7.34	0.194	0.04
CP ^1^	0.92	0.94	0.026	0.43
NDF ^1^	2.93	3.14	0.083	0.02
ADF ^1^	1.28	1.42	0.036	0.002
Starch	1.47	1.70	0.043	<0.001
Total-tract apparent digestibility (g/kg)				
DM	670	636	11.9	0.02
OM	695	664	10.9	0.02
CP	645	621	16.1	0.16
NDF	582	535	12.8	0.01
ADF	520	468	33.0	0.15
Starch	877	874	4.76	0.59
ADG ^1^ (kg/d)	1.19	1.15	0.093	0.37

^1^ DM, dry matter; OM, organic matter; CP, crude protein; NDF, neutral detergent fiber; ADF, acid neutral detergent fiber; ADG, average daily gain.

**Table 6:** Dissolved gases and fermentation end-products in the rumen of beef cattle fed corn stover after fresh-corn harvested (CF) or corn stover after ripe-corn harvested (CR) silage diet (n = 7).

Items	Diets		Sampling Time (h)		SEM	*p*-Value		
	CF	CR	0	2.5		Diet	Time	Diet × Time
Dissolved gases								
Hydrogen (μM)	0.50	0.84	0.88	0.46	0.132	0.21	0.12	0.43
Methane (mM)	0.71	0.77	0.80	0.68	0.026	0.25	0.03	0.19
pH	6.89	6.82	6.93	6.78	0.013	0.02	<0.001	0.008
Ammonia-N (mM)	7.51	6.87	3.98	10.4	0.426	0.46	<0.001	0.04
Total VFA ^1^ (mM)	67.0	62.2	56.6	72.6	2.52	0.14	<0.001	0.70
Molar percentage of individual VFA (mol/100 mol)								
Acetate	65.4	64.9	67.6	62.7	0.296	0.48	<0.001	0.87
Propionate	19.5	19.3	17.4	21.4	0.286	0.65	<0.001	0.49
Butyrate	10.7	11.3	10.7	11.4	0.330	0.18	0.12	0.20
Valerate	1.3	1.3	1.0	1.6	0.052	0.61	<0.001	0.92
Isobutyrate	1.3	1.3	1.4	1.2	0.015	0.03	<0.001	0.17
Isovalerate	1.8	1.8	1.9	1.8	0.039	0.69	0.10	0.45
Acetate to propionate ratio	3.42	3.45	3.91	2.95	0.184	0.80	<0.001	0.68
Estimated net hydrogen production relative to the amount of total VFA produced (mol/mol)								
	1.32	1.33	1.39	1.26	0.013	0.57	<0.001	0.34

^1^ VFA, volatile fatty acid.

**Table 7:** Selected microbial groups (log_10_ gene copies per mL rumen content) in the rumen of beef cattle fed corn stover after fresh-corn harvested (CF) or corn stover after ripe-corn harvested (CR) silage diet (n = 7).

Items	Diets		SEM	*p*-Value
	CF	CR		
Bacteria	12.1	12.0	0.030	0.17
Protozoa	10.7	10.4	0.042	0.01
Methanogens	8.73	8.57	0.043	0.09
Fungi	7.95	7.43	0.073	0.01
Selected groups of bacteria				
* Ruminococcus albus*	8.94	8.72	0.060	0.10
* Ruminococcus flavefaciens*	8.69	8.54	0.053	0.18
* Fibrobacter succinogenes*	9.77	9.58	0.027	0.004

**Table 8:** Alpha diversity indexes of bacterial community, as determined by 16S rRNA gene sequencing in the rumen content of beef cattle fed corn stover after fresh-corn harvested (CF) or corn stover after ripe-corn harvested (CR) silage diet (n = 7).

Items	Diets		SEM	*p*-Value
	CF	CR		
Alpha diversity indexes				
Chao1	1956	2083	67.4	0.36
Species	1384	1460	40.8	0.37
Shannon	8.64	8.64	0.101	0.97
Coverage	0.966	0.963	0.001	0.26

## Data Availability

The data presented in this study are available in this article and the Supplementary Materials.

## Supplementary Materials

The following are available online at https://www.mdpi.com/article/10.3390/ani12101248/s1, Table S1: The bacterial phyla and genera (>1% abundance) as determined by 16S rRNA gene sequencing in the rumen content of beef cattle fed corn stover silage after fresh-corn harvested (CF) or corn stover silage after ripe-corn harvested (CR) diet (n = 7).
