# Supplementary material for: Comparisons of Corn Stover Silages after Fresh- or Ripe-Corn Harvested: Effects on Digestibility and Rumen Fermentation in Growing Beef Cattle
Source: Animals (Basel). 2022 May 13;12(10):1248. doi: 10.3390/ani12101248 (PMC9137847; doi:10.3390/ani12101248)
Supplement: Supplementary file 1 [file animals-12-01248-s001.zip › animals-1697751-supplementary.pdf]

Table S1. The bacterial phyla and genera (> 1% abundance) as determined by 16S rRNA gene sequencing in the rumen content of beef cattle fed corn stover silage after fresh-corn harvested (CF) or corn stover silage after ripe-corn harvested (CR) diet (n = 7).

**Items**[1]	**Diets**		**SEM**	***p*-value**
	**CF**	**CR**		
p_Bacteroidetes	56.7	58.1	1.15	0.06
g_*Prevotella*_1	14.5	15.8	1.13	0.48
g_*Rikenellaceae*_RC9_gut_group	12.7	11.0	0.808	0.22
g_*Prevotellaceae*_UCG-003	2.48	2.69	0.268	0.22
g_*Prevotellaceae*_UCG-001	2.63	2.48	0.220	0.95
p_Firmicutes	35.3	32.8	1.15	0.06
g_*Succiniclasticum*	4.75	4.71	0.396	0.65
g_*Ruminococcaceae*_UCG-011	2.49	2.09	0.179	0.41
g_*Ruminococcaceae*_NK4A214_group	2.34	1.76	0.154	0.04
g_*Ruminococcaceae*_UCG-014	1.93	1.91	0.140	0.75
g_*Saccharofermentans*	1.93	1.71	0.106	0.34
g_*Ruminococcus*_1	1.80	1.42	0.094	0.06
g_*Ruminococcaceae*_UCG-010	1.46	1.45	0.057	0.85
g_*Christensenellaceae*_R-7_group	1.38	1.26	0.087	0.65
g_*Lachnospiraceae*_AC2044_group	1.15	1.13	0.072	0.57
p_Fibrobacteres	1.60	1.67	0.128	0.65
g_*Fibrobacter*	1.57	1.64	0.128	0.65
p_Patescibacteria	1.61	2.02	0.149	0.18
g_*Candidatus_Saccharimonas*	1.27	1.64	0.141	0.22
p_Proteobacteria	1.27	1.45	0.122	0.34
p_Kiritimatiellaeota	0.92	1.14	0.082	0.14

[1]p = phylum, g = genus.
